# A novel hybrid explainable artificial intelligence modelling approach for smart manufacturing

**DOI:** 10.1007/s00170-025-17157-4

**Published:** 2026-02-04

**Authors:** Puthanveettil Madathil Abhilash, Xichun Luo, Qi Liu, Yi Qin

**Affiliations:** Centre for Precision Manufacturing, DMEM, University of Strathclyde, Glasgow, G1 1XJ UK

**Keywords:** Artificial intelligence, Smart manufacturing, Explainable artificial intelligence, Symbolic regression, Hybrid physics-based model

## Abstract

Modelling complex manufacturing processes presents significant challenges related to accuracy and explainability. Physics-based models, while interpretable and generalizable, often suffer from reduced accuracy due to simplifications and incomplete system understanding. On the other hand, purely data-driven models are typically more accurate but lack transparency, limiting their trust and adoption in critical manufacturing applications. Existing hybrid approaches attempt to address these issues but often retain black-box AI components that compromise interpretability. In this study, we propose a novel hybrid modelling framework that intrinsically integrates physics-based models with explainable AI, to correct for modelling inaccuracies. This approach offers both high accuracy and transparent, traceable decision-making. Its effectiveness is demonstrated through a case study predicting the real-time position of cutting tools from accelerometer signals during ultra-precision diamond turning.

## Introduction

Accurate predictive modelling of complex manufacturing processes has been an open scientific challenge for quite some time. Despite the significant computational advancements, the complexity and high dimensionality of data have made purely physics-based modelling extremely challenging. This is typical in the case of smart manufacturing processes involving interconnected systems and sensors generating vast amounts of real-time data. While physics-based models offer fundamental understanding, they often struggle to capture the nuances of smart manufacturing dynamics involving missing, noisy data and larger levels of approximations [1].

To address the limitations of purely physical models, data-driven approaches like Artificial Intelligence (AI) modelling have been widely applied, especially in applications like condition monitoring, anomaly detection, and process control, owing to their superior capability in identifying hidden patterns and complex correlations even within complex and noisy data [2, 3]. However, many of these advanced machine learning (ML) models offer poor interpretability of the extracted information. Moreover, despite their ability to accurately fit the data, data-driven models have limited generalization capabilities due to their physical inconsistency and biases.

To address the limitations of both modelling paradigms, we propose a novel hybrid framework that integrates intrinsically explainable AI into physics-based modelling to compensate for its inherent errors. This allows for accurate, interpretable predictions of complex manufacturing phenomena, as demonstrated in a real-time tool position tracking case study.

## Literature review

Predictive modelling of complex manufacturing processes traditionally relies on two dominant paradigms: physics-based models and data-driven approaches. While both have their merits, each faces inherent limitations that limits their effectiveness in the context of modern smart manufacturing systems. Physics-based models are grounded in established physical laws and domain knowledge, offering high interpretability and generalizability. These models simulate system behavior through mathematical formulations derived from first principles, making them valuable for scientific understanding and physical consistency [4–6]. However, their performance often deteriorates in real-world manufacturing scenarios due to the complexities of high-dimensional, nonlinear systems and imperfect knowledge of process physics. Furthermore, these models require significant computational resources and are sensitive to simplifications and assumptions, leading to reduced predictive accuracy in the presence of noise, uncertainty, or partial observability of system dynamics [7–9].

In contrast, data-driven models, particularly AI, excel in capturing nonlinear correlations and hidden patterns in complex, multivariate datasets. Their adaptability and predictive power make them suitable for various manufacturing tasks, including anomaly detection, condition monitoring, and process control [10–14]. However, their major drawback lies in their lack of transparency. Deep learning models are often treated as “black boxes”, offering limited insights into the underlying causal mechanisms of the system. This opacity raises concerns in safety-critical and high-precision manufacturing domains, where explainability and trust in model decisions are essential [15–17]. Moreover, data-driven models are prone to overfitting and lack generalization when exposed to new operating conditions or data distributions outside the training set. They also struggle to integrate domain knowledge, often leading to physically inconsistent predictions [18, 19].

These limitations emphasise the need for an integrated modelling approach that can combine the scientific rigour of physics-based models with computational efficiency and predictive accuracy of data-driven methods, while also addressing the growing demand for explainability in intelligent manufacturing systems. In this context, hybrid modelling approaches have emerged as a promising alternative [20]. Several recent studies have explored methods to fuse physical knowledge with machine learning, particularly in smart manufacturing contexts. A common strategy involves physics-informed machine learning (PIML), where physical constraints or governing equations are embedded into ML training through tailored loss functions, regularization terms, or architecture designs. For instance, Wang et al. [19] developed a physics-guided neural network for tool wear prediction that incorporated a physics-based loss term to improve consistency and generalization. Similarly, Zhao et al. [18] used a physics-informed latent variable model to predict part deformation, enhancing both accuracy and physical coherence.

Another class of hybrid approaches includes ML-assisted simulation models, where machine learning techniques are used to augment or accelerate computational physics solvers. These models can either act as surrogates to reduce simulation cost or learn correction functions to adjust for model deficiencies. Willard et al. [21] and Wang et al. [22] provide extensive reviews on such integration strategies, highlighting the potential of hybrid approaches across manufacturing, climate science, and energy systems.

In manufacturing, hybrid physics–data modelling has been extensively explored for tool wear and process condition monitoring. Gao et al. [23] proposed a hybrid physics data-driven model-based fusion framework for machining tool wear prediction, in which physical wear models and local sensor features are combined to compensate for the limitations of each component. Similarly, Pashmforoush et al. [24] developed a physics-informed tool wear prediction framework for turning, integrating a thermo-mechanical force model with machine-learning corrections to improve robustness across operating conditions. More recently, dual-knowledge embedded hybrid models have been introduced that augment limited labelled data with physics-consistent synthetic samples and embed physical constraints directly into the learning process for tool wear prediction [25]. These studies clearly demonstrate the growing interest in hybrid physics–ML tools for monitoring and compensating machining-related degradation mechanisms.

The hybrid techniques have also been applied to tasks such as anomaly detection, fault diagnosis, and process optimization. Notably, Wilhelm et al. [26] present an overview of fault detection systems that integrate physical, data-driven, and knowledge-based models into unified frameworks. Such architectures show promising performance, especially under conditions of limited data availability or partial physical understanding.

**Fig. 1:**
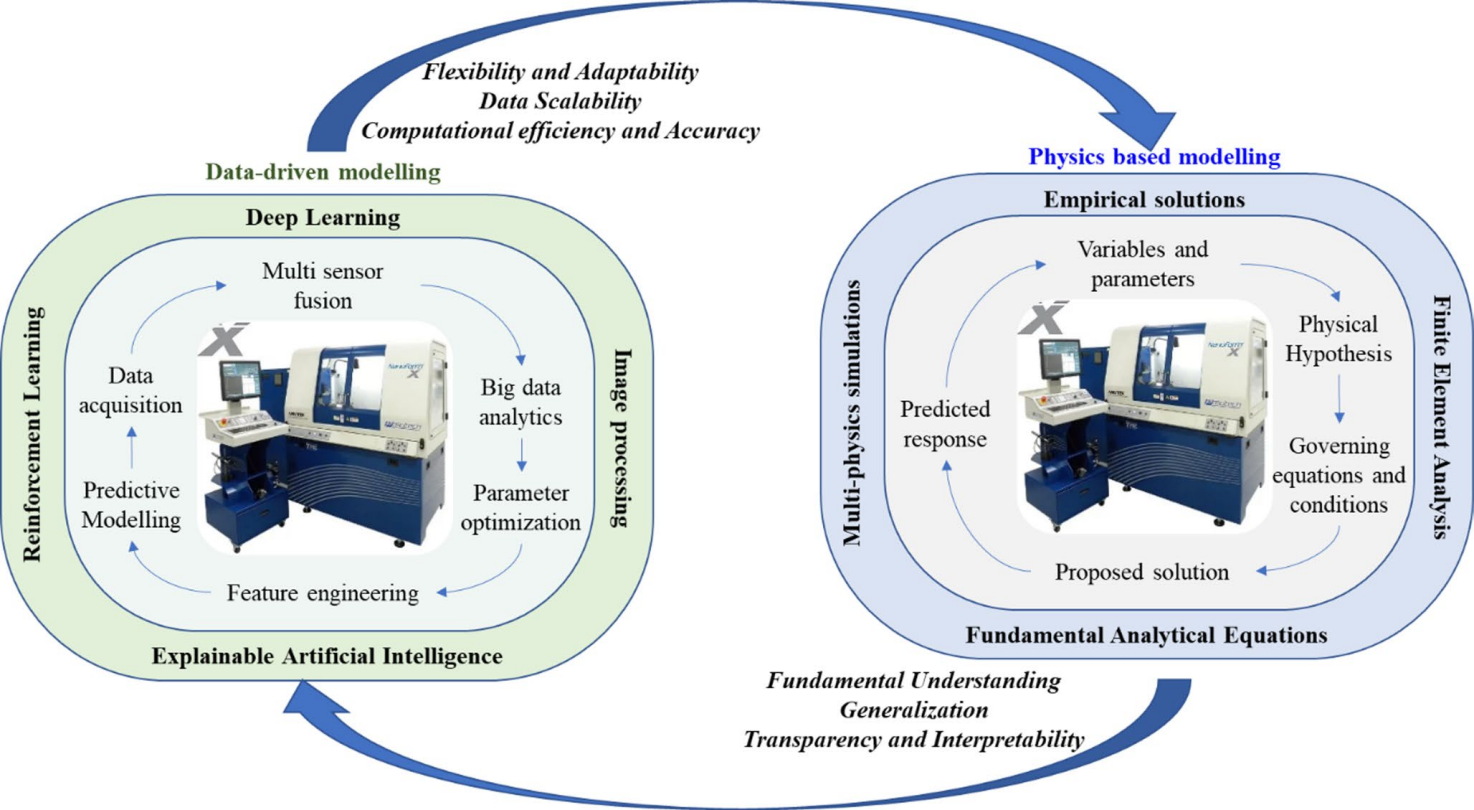
Concept of combined advantages of a hybrid physics-based data-driven model

Figure 1 illustrates the conceptual framework behind hybrid modelling, where the strengths of both physics-based and data-driven models are combined. In such configurations, data-driven models correct for limitations in physical models (such as approximations and incomplete physics), while physical models constrain data-driven learners, enhancing consistency and robustness. The hybrid strategy thus offers an effective balance by delivering higher predictive accuracy without sacrificing scientific interpretability.

Though the concept is promising, most existing hybrid models face a critical challenge: the trade-off between accuracy and interpretability. Although many frameworks improve predictive performance, they typically rely on black-box neural networks or ensembles, which diminish the overall transparency of the hybrid architecture. The complex computational architecture introduce opacity, reducing engineers’ ability to validate predictions, understand failure modes, or extract actionable insights. This limitation is especially problematic in industrial settings, where regulatory and safety requirements demand explainable and traceable model behaviour [27, 28]. This lack of interpretability also limits generalisation, as models may be overfit without offering a mechanistic rationale that supports extrapolation or domain transfer. The next-gen hybrid modelling approach needs to maintain interpretability without sacrificing accuracy, which we aim to achieve through the integration of explainable AI (XAI) techniques.

Recent research in XAI aims to address the shortcomings of black-box ML by offering techniques to improve model transparency. These can be broadly categorized as: Post-hoc XAI, where interpretability is applied retrospectively using tools like SHAP or LIME to explain predictions made by opaque models [29, 30], Intrinsic XAI, where the model is inherently interpretable, often through analytical expressions, decision trees, or sparse models [17]. While post-hoc XAI is useful, it often fails to deliver true model understanding or insight into learned representations, especially when the base learner is inherently non-linear or high-dimensional. This makes intrinsically interpretable models more attractive for integration into hybrid frameworks, as they preserve the traceability and semantic clarity of physical models.

Despite its potential, XAI, especially intrinsic forms like symbolic regression, remains underexplored in the context of hybrid modelling for manufacturing. Recent work has begun to apply symbolic regression in manufacturing-related settings. Asadzadeh et al. [31] used symbolic regression to construct hybrid semiparametric models for metal sheet bending, showing that partially physics-guided SR can outperform purely data-driven approaches while retaining interpretability. Kabliman et al. [32] employed symbolic regression to derive constitutive models for plastic deformation that can be embedded into finite-element simulations, demonstrating the suitability of SR for generating physically meaningful, simulation-ready equations. However, these studies do not offer insights into their use within a more general modelling context.

The review of existing modelling approaches reveals a distinct gap in the landscape of smart manufacturing: while hybrid models have advanced the predictive performance of process modelling, they often do so at the expense of interpretability. As a result, many solutions remain unsuitable for applications that demand transparency, physical consistency, and scientific insight. Current hybrid strategies either: (a) Rely on black-box neural networks for error correction or pattern learning, which limits their usefulness in high-precision industrial environments; or (b) fail to integrate domain knowledge meaningfully, leading to physically inconsistent or overfitted predictions.

This study seeks to fill that gap by proposing a hybrid physics-based XAI modelling approach, wherein:

- A physical model serves as the foundation for process simulation,
- The inherent modelling errors (i.e., deviations from ground truth) are quantified as correction factors,
- These correction factors are predicted using an XAI model, producing analytic expressions that retain interpretability.

The novelty of the proposed framework lies in its integration of physics and explainable machine learning, enabling a model that is not only more accurate but also transparent, generalizable, and physically coherent. This is particularly valuable in manufacturing settings that generate complex, noisy sensor data and demand real-time decision-making with traceable logic. By embedding interpretability directly into the data-driven component of the hybrid system, this approach introduces a new paradigm for developing trustworthy, physically consistent, AI-enabled digital twins in advanced manufacturing.

## Methodology

### Symbolic regression using qlattice

Symbolic regression is a technique that searches the space of mathematical expressions to identify analytic relationships between inputs and outputs. It is widely regarded as one of the most effective XAI tools due to its ability to express model logic in closed-form Eqs [33, 34].

Several families of symbolic regression algorithms have been proposed, including genetic programming–based methods, physics-inspired approaches such as AI Feynman, and recent sparse regression and operator-learning techniques. While genetic programming can, in principle, discover rich analytic forms, it is often computationally expensive, prone to “bloat” (unnecessarily large expressions), and sensitive to hyperparameter tuning, especially for noisy, medium-sized industrial datasets. Physics-informed ML frameworks and physics-informed neural networks, on the other hand, typically embed governing equations or physical constraints directly into deep network training. Although powerful, these methods usually produce black-box neural architectures rather than closed-form expressions and require detailed prior knowledge of the governing PDEs for each new application.

In this study, we employ Qlattice, a quantum-inspired symbolic regression engine that efficiently identifies interpretable mathematical models. It represents model structure using Qgraphs - spatial path representations of functional relationships - and simulates Feynman’s path integral formulation to guide the search process [35–37]. In contrast to other symbolic regression approaches, Qlattice offers a sample-efficient symbolic regression engine with built-in complexity control and feature selection, generating compact analytical expressions that are well suited for use as correction models in hybrid physics-based frameworks. This balance of computational efficiency, robustness to noise, and intrinsic interpretability motivated our choice of Qlattice in the present work. Key features of the Qlattice approach include:

- User-defined operator sets, including basic arithmetic and domain-specific unary functions (e.g., trigonometric, Gaussian, logarithmic).
- Loss function customization (RMSE used here).
- Automatic complexity control, where model complexity is penalized to ensure interpretability.
- Feature selection, where only the most relevant features are retained in the final expression.

This setup allows Qlattice to generate equations that not only match observed data accurately but also highlight meaningful physical relationships, critical for both model validation and downstream decision-making. To ensure reproducibility, the Qlattice model was trained using a fixed random seed (seed = 42), ensuring repeatability of the symbolic search process. The training process was executed for 10 epochs (without any automatic convergence-based early stopping). The built-in Qlattice complexity penalty parameter was left at its default setting (λ = 1.0), which penalizes model complexity based on the number of operators and selected features. Uncertainty in the symbolic model was quantified by reporting the MAE and RMSE.

### Overview of hybrid modelling approach

The overall methodology of embedding XAI in physical models has the following key steps.

**Fig. 2:**
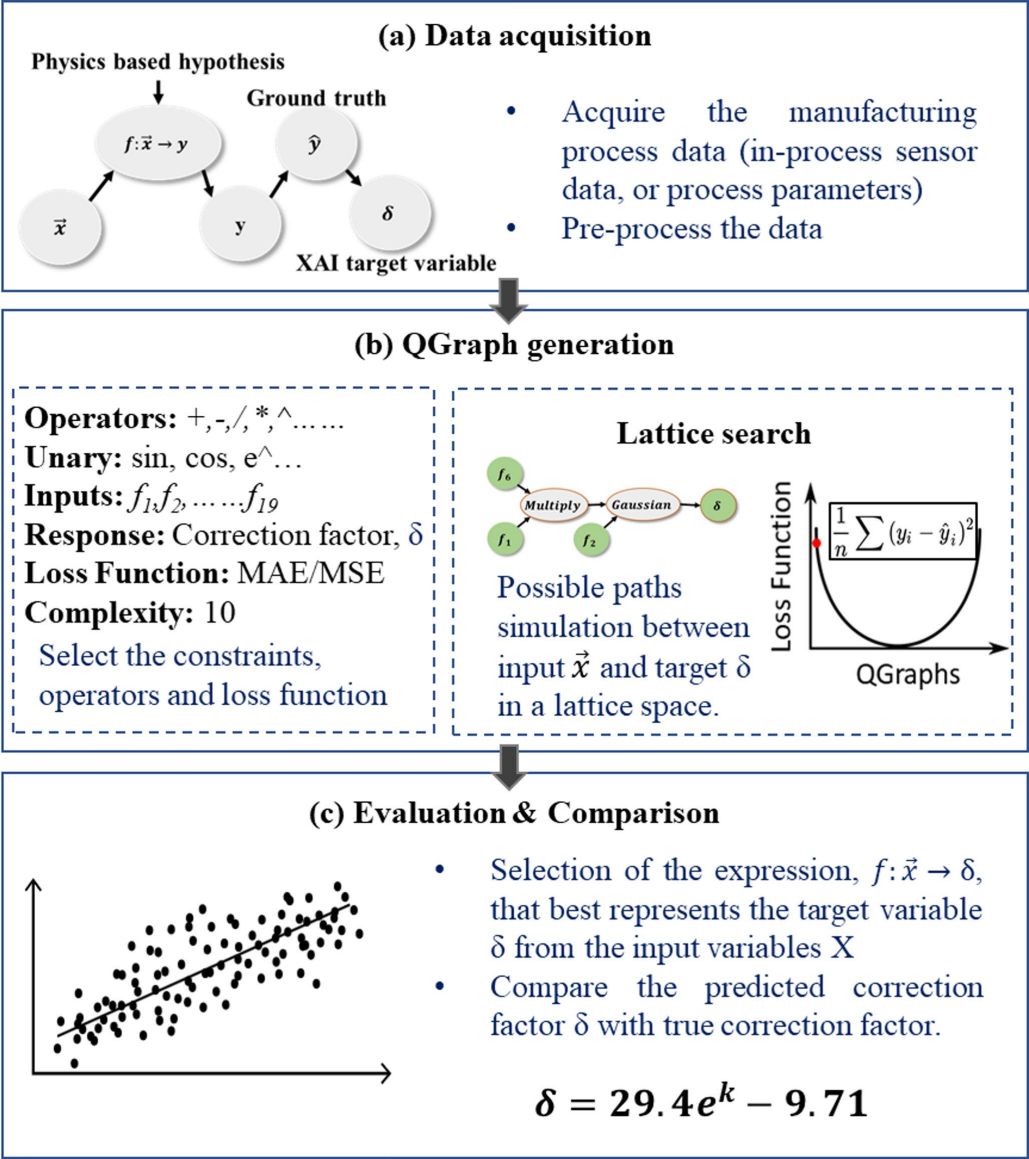
Role of XAI in a hybrid physics-based model

1. *Physical modelling*: The core of the hybrid model needs to centre around the fundamentals of process physics to ensure overall physical interpretability, scalability and generalisability. This implies that the foundational model of the hybrid approach is the physics-based model. In this regard, initially, the physics-based model is developed based on the physical, empirical or mathematical understanding of the underlying physics.
2. *Computing correction factor*: This step quantifies the physical modelling errors caused by system complexity, computational complexities, partial understanding of underlying physics, approximations and uncertainties. The cumulative inherent errors of the physics-based models are estimated based on the ground truth data. From a hybrid modelling perspective, these errors are defined, and from here on mentioned, as the correction factor (δ) required to improve the performance of physics-based models.
3. *XAI modelling*: This step uses a data-driven approach to predict the correction factor, δ. Here, symbolic regression, a prominent XAI approach, is employed to predict the correction factor. The technology ensures the preservation of the overall physical interpretability even after integrating the data-driven and physics-based models. The concept of XAI-driven prediction of the correction factor is shown in Fig. 2. This is a significant departure from the existent hybrid modelling approaches, where the opaqueness of ML models negatively affects the overall transparency and interpretability of the hybrid model.

(4) *Model integration*: Model integration involves combining the physics-based and XAI models to form a hybrid model that performs better than a purely physics-based model. Overall, the generic model integration approach is represented mathematically as follows:

The physical model $\:{f}_{p}$ that correlates the input parameters $\:\overrightarrow{x}$ and target variable $\:y$ is given as.

1 $$f_p:\overrightarrow x\rightarrow\:y$$

If $\:\widehat{y}$ is the ground truth data from experiments or better accuracy measurement, then the correction factor $\:\delta\:$ is given by,

2 $$\delta=\widehat y-f_p\left(\overrightarrow x\right)$$

Now, the XAI model $\:{f}_{XAI}$ which aims to correlate the input vector $\:\overrightarrow{x}$ and $\:\delta\:$ is represented as.

3 $$f_{XAI}:\overrightarrow x\rightarrow\delta\:$$

Finally, the hybrid physics-based XAI model, $\:{f}_{H}$ is

4 $$\:{f}_{H}:\overrightarrow{x}\to\:y\left(\overrightarrow{x}\right)\mathrm{+}{f}_{XAI}\left(\overrightarrow{x}\right)$$

5 $$\:{f}_{H}:\overrightarrow{x}\to\:y\:\left(\overrightarrow{x}\right)\text{}{\mathrm{+}\delta\:}_{XAI}$$

(5) *Validation and verification*: As the final step, the hybrid model is validated against the ground truth data.

**Fig. 3:**
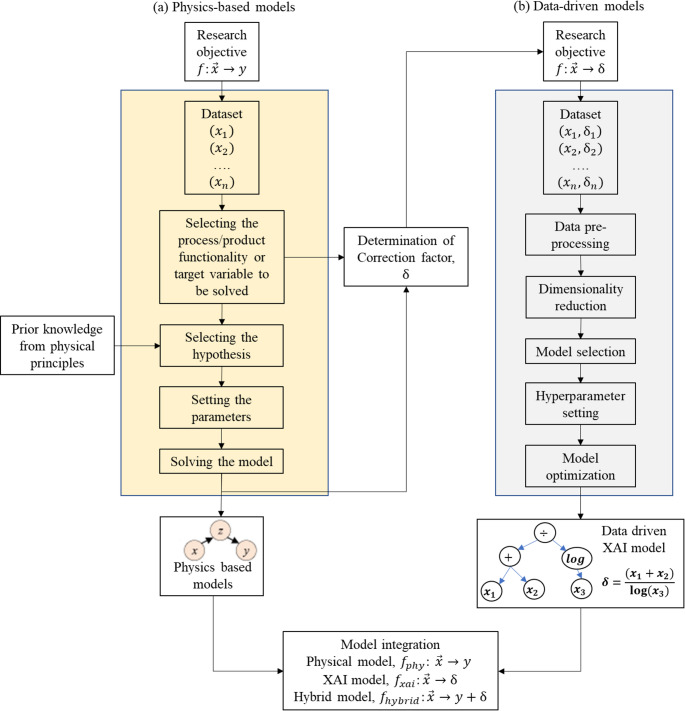
A generic framework for hybrid physics-based XAI modelling

In summary, the hybrid model uses a physics-based model as its foundation and subsequently, utilizes the XAI to apply the correction factor to enhance its predictive performance. The overall methodology is given in the Fig. 3.

## Experiment details

The XAI model is developed on Python programming language version 3.9.12. XAI model Qlattice from the Feyn library is executed within a web-based Jupyter Notebook environment on the Anaconda3 Python distribution platform (version Conda 22.9.0). For the XAI modelling, the 80:20 train-test data split ratio is followed. Also, to more rigorously evaluate generalisation and robustness, we performed K-fold cross-validation (K = 5) on the same dataset. In the K-fold procedure, the data were partitioned into five disjoint folds; in each iteration, four folds were used for training and the remaining fold for testing, and this process was repeated until each fold had served as the test set once. The final cross-validated performance is reported as R² and RMSE across all folds. Every other computation including the physics-based modelling, development of the decision tree regressor, and the final model integration is done in MATLAB.

The proposed approach is demonstrated by considering the case study of modelling real-time displacements from accelerometers, in pursuit of predicting the precise tool tip position for ultra-precision machines.

### Case study: accurate TCP position tracking during ultra-precision diamond turning

Slow tool servo (STS) ultra-precision diamond turning has been proven as a capable approach towards the low-cost manufacture of free-form, rotationally asymmetric optics. In this process, the machine slide Z-axis moves periodically in sync with spindle rotation to achieve complex surfaces. However, during STS operation, the actual position of the Tool Center Point (TCP) can vary significantly from its intended position, especially at higher frequency z-axis motions due to the machine dynamics including the vibrations, jerk, and resonance. Such deviations, often called dynamic errors can cause form inaccuracies and poor surface integrity of machined parts. In literature, dynamic errors are defined as the difference between the actual motion of a system and the desired/expected motion. The primary reason behind dynamic errors is reported to be the vibrations and oscillations of the system’s flexible elements outside the servo loop [38]. Due to this, the machine’s in-built encoders will not be able to capture the true position of the TCP.

In this context, an accelerometer-based position measurement system for ultra-precision machines is highly relevant. Such an approach can provide a more accurate evaluation of real-time tool displacements being able to account for the motions outside the servo loop when mounted close to TCP. However, extracting the real-time displacements from acceleration data remains quite a challenge.

The experimental setup includes a PCB 356B triaxial ICP accelerometer mounted on the tool holder near the TCP of a 3-axis diamond tuning machine to acquire real-time accelerometer signals. For training and validation of the proposed approach, the true real-time (ground truth) position data at TCP is measured via a 1 nm resolution capacitance probe (CP8.0–2.0.0-2.0, IBS Precision Engineering). The considered tool motion is sinusoidal in the Z-axis with varying amplitudes and frequencies, as detailed in Table 1. The signals acquired are command, encoder data, capacitance probe data, and accelerometer data using a data acquisition system consisting of a PCI-6259 DAQ (National Instruments, USA) and a linear digital amplifier (Aerotech Ndrive HPe). All analyses are carried out on a Windows 11 desktop PC, using MATLAB 2022a and Python.

**Table 1:** Test details for sinusoidal command signal of the form, $\:ASin\left(\omega\:t\right)$

Amplitude, A (µm)	4	5	6	7	8	9	10	11
Frequency, ω (Hz)	10	15	20	25	30	35	40	

## Results and discussion

The proposed hybrid XAI modelling approach is demonstrated by modelling real-time displacement predictions from online accelerometer sensor signals, the results of which are discussed next.

### Modelling real-time TCP position using accelerometers

For the training data generation, the command amplitudes are varied from 4 μm to 11 μm in steps of 1 μm, and frequencies are varied from 10 Hz to 40 Hz in steps of 5 Hz. During this motion, the data captured in real-time are (a) the command signals (b) the capacitance probe data (ground truth - actual position data) (c) machine encoder measurements (d) accelerometer signals, all in the z-axis.

### Dynamic error measurement

**Fig. 4:**
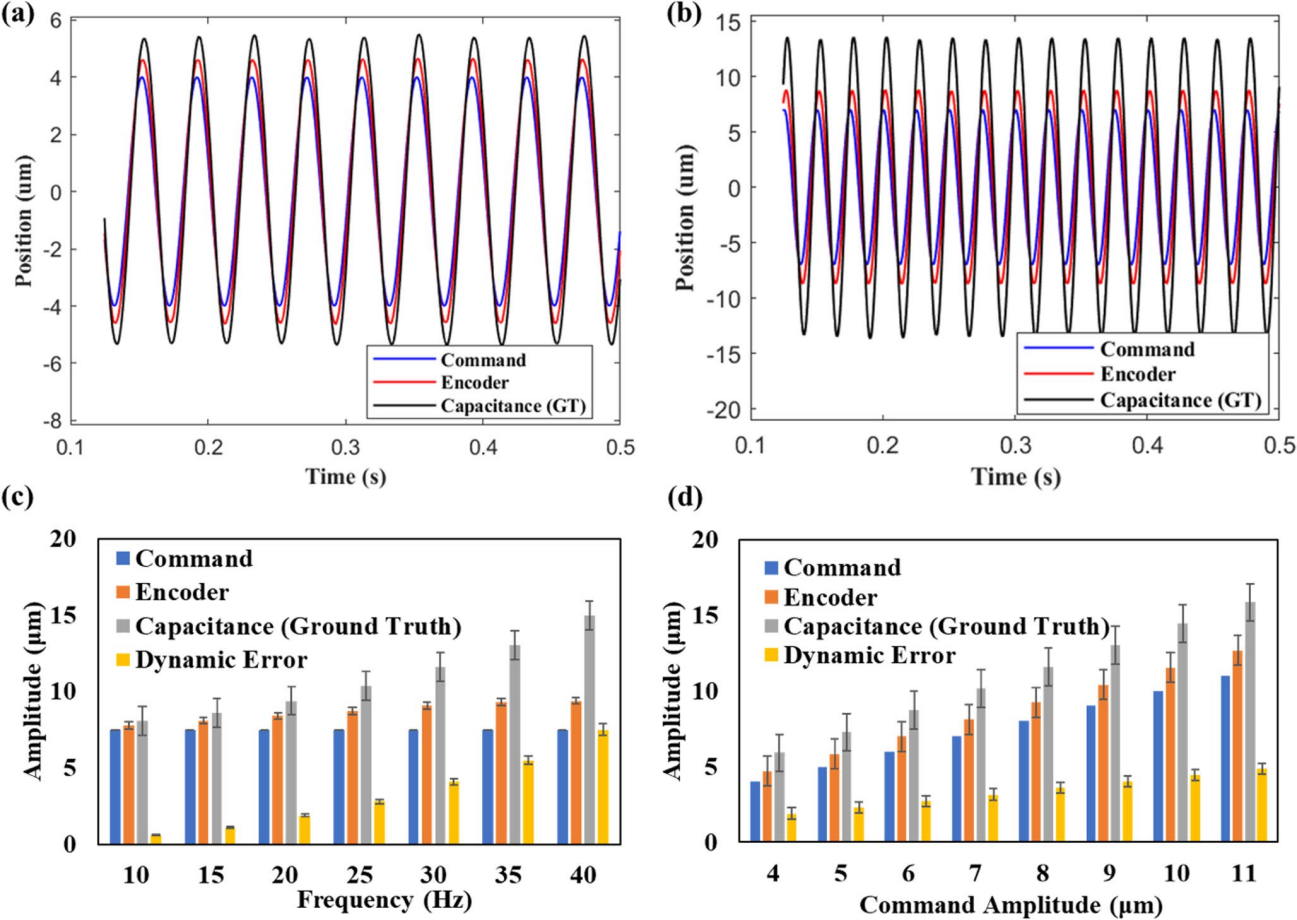
Comparing the TCP position data at (**a**) 10 Hz and (**b**) 40 Hz; average variation of peak amplitudes at different (**c**) frequencies and (**d**) amplitudes of the command signal

The command signals are compared with encoder measurements and capacitance sensor readings during sinusoidal cutting tool motion. Figures 4(a) and 4(b) compare these three signals during sinusoidal command motions of frequencies 10 Hz and 40 Hz respectively. The peak amplitudes of the sinusoidal motion captured by the capacitance sensor and machine encoder are higher than those of the command, and this error is more pronounced at 40 Hz than at 10 Hz. This deviation, referred to as dynamic error, is defined as the difference between the true and desired positions. Dynamic error arises from the vibration, deflection, and jerk phenomena of the machine’s flexible elements, and is hence more prominent at higher operational frequencies. It is worth noting that the machine’s default encoder measurements are not accurate enough and lag behind the capacitance probe in tracking the true TCP position. Figures 4(c) and 4(d) compare the average peak amplitudes of the sinusoidal position data measured by command, encoder, and capacitance probe at varying frequencies and amplitudes respectively. Dynamic error is observed as a significant factor, increasing with both command amplitude and frequency, thus causing the TCP position to deviate from its intended values.

The presence of dynamic errors highlights the need for an alternative approach using accelerometers to precisely compute the TCP during STS. The classical physics-based approach involves double integrating the acceleration signals to obtain displacement [39], the results of which are discussed next.

### Physics-based approach

The physics-based method employs double integration of accelerometer signals to calculate the displacement, based on Newtons laws of motion correlating acceleration, velocity and displacement [40]. Based on the physical interpretation of acceleration, integrating it once will provide the velocity, and a further integration gives the displacement. In the past, the double integration approach has been successfully demonstrated in the fields of structural monitoring [41, 42] and seismology [43, 44]. Recently, the method has also been expanded to the machine tool motion control [39, 45].

In this study, the displacement signals are extracted by double integrating the band-pass filtered accelerometer data. Filtering mitigates the noise and unwanted artefacts. The displacement $\:d\left(t\right)$ at the TCP can be obtained through double integration of the bandpass-filtered accelerometer signals $\:{a}_{F}\left(t\right)$ as expressed through Eq. (6) and Eq. (7). Here$\:v\left(t\right)$ is the velocity at time t

6 $$v\left(t\right)=\int\;a_F\left(t\right)\hspace{0.17em}dt$$

7 $$d\left(t\right)=\int\:v\left(t\right)\hspace{0.17em}dt$$

**Fig. 5:**
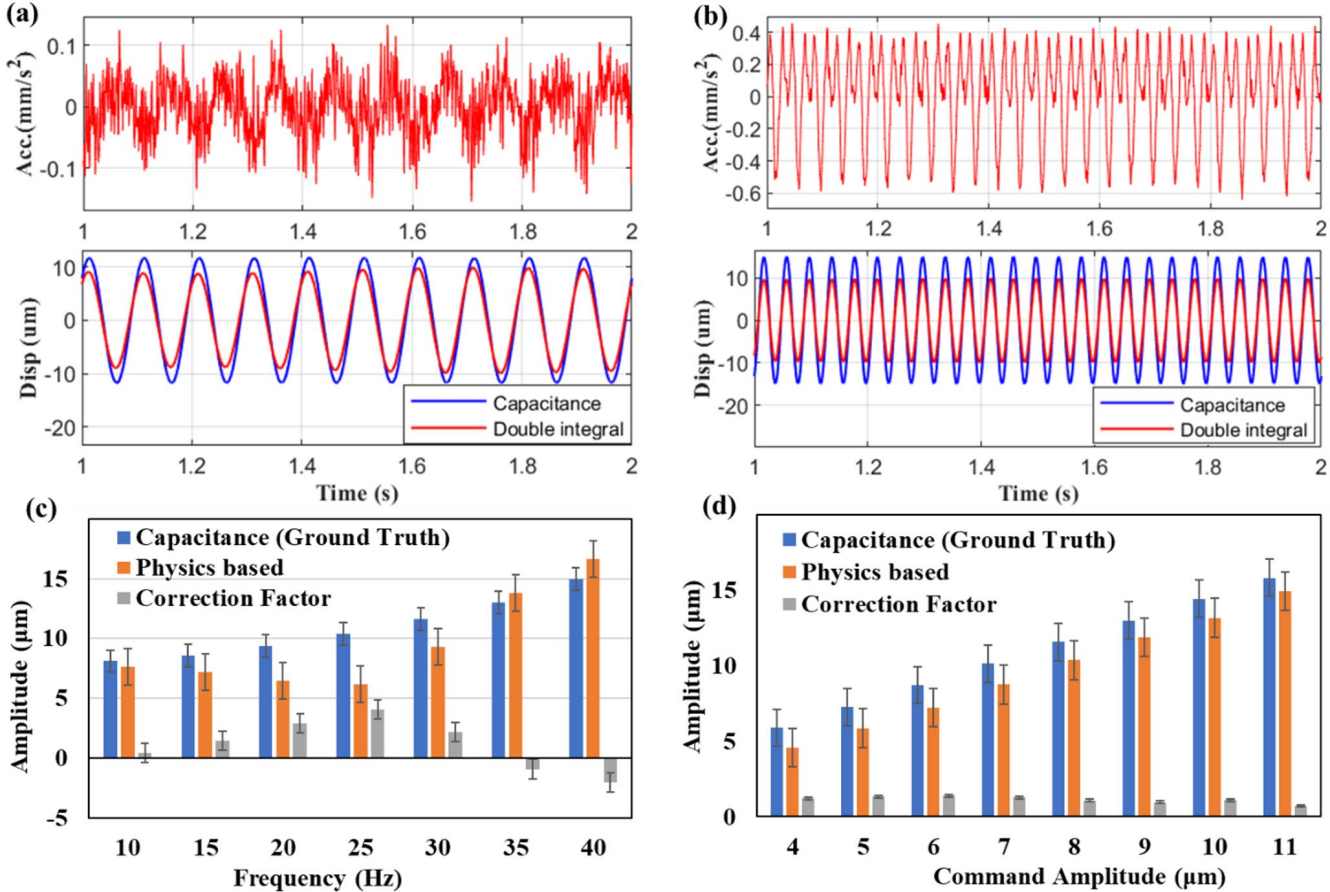
Physics-based vs. actual positions at (**a**) 10 Hz (**b**)25 Hz; correction factor variations concerning (**a**) frequencies (**b**) amplitudes

Figure 5 compares the double-integrated displacement signals against the actual displacement at 10 Hz (Fig. 5 (a)) and 25 Hz (Fig. 5 (b)). Upon comparison, the method is not accurate enough and the inaccuracies are more prominent at higher frequencies. The inaccuracies of the physics-based approach can stem from the noise amplification, drifts and effects of dynamic acceleration.

To enhance the accuracy of the double integration method, it is necessary to define and model its inherent error. The proposed approach defines the physical model errors as the correction factor (δ), which is the amount by which the physical model must be adjusted so that it approaches the ground truth. Figure 5 (c) and Fig. 5 (d), quantify and compare the average correction factor against command frequencies and amplitudes respectively.

### Hybrid physics based XAI model

**Fig. 6:**
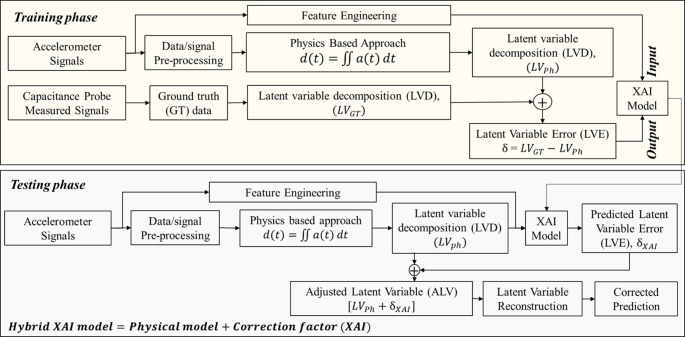
Hybrid physics-based XAI modelling of displacement from accelerometers

The basic concept of the proposed approach is to model the correction factor using XAI to improve the physical model predictions. For the current case, the methodology of modelling and accounting for the correction factor is shown in Fig. 6. The process involves 3 stages: latent variable decomposition (LVD) from the physical model, correction using XAI, and finally the reconstruction. LVD is defined as identifying hidden (unobservable) variables from the raw signals, which here is the peak amplitude of displacement signals. As mentioned in the previous section, the latent variable of the physics-based model ($\:LV{D}_{Ph}$) is compared against the latent variable of ground truth ($\:LV{D}_{GT}$) signal. The correction factor, δ, is the latent variable error (LVE), obtained as the difference between $\:LV{D}_{GT}$ and $\:LV{D}_{Ph}$. XAI is trained to predict δ based on the extracted features from accelerometer signals. During the testing phase, the predicted correction factor ($\:{\delta\:}_{XAI}$), based on unseen accelerometer signals, is integrated into the $\:LV{D}_{Ph}$ to obtain an adjusted latent variable ($\:ALV$), which is subsequently used to reconstruct the accurate position data of TCP. Symbolic regression modelling of δ is elaborated in the next section.

In the context of sinusoidal slow-tool-servo (STS) motion, the displacement signal can be well represented by a dominant latent variable, the peak amplitude of the periodic response. This variable captures the fundamental dynamic behaviour of the Z-axis displacement and directly reflects the physical deviation introduced by the machine dynamics. Therefore, the displacement signal x(t) obtained from both the physical model and the ground truth can be decomposed as

8 $$x\left(t\right)=Asin\left(\omega t+\phi\right)$$

where A is the latent variable (peak amplitude), ω is the command frequency, and φ is the phase.

The LVD step extracts the peak amplitude from both the physical-model output ($\:{A}_{phys}$) and the ground-truth signal ($\:{A}_{true}$) The correction factor is then defined as:

9 $$\:\delta=\:A_{true}-A_{phys}$$

Once the XAI model predicts the correction factor $\:\widehat{\:\delta}$ for an unseen accelerometer signal, the corrected latent amplitude is computed as:

10 $$A_{corr}=A_{phys}+\widehat \delta$$

The corrected displacement signal is then reconstructed by replacing the amplitude in the sinusoidal form:

11 $$\widehat x\left(t\right)=A_{corr}sin\left(\omega t+\phi \right )$$

This reconstruction preserves the temporal structure and phase characteristics of the physics-based displacement while correcting only the amplitude component where the majority of physical-model error occurs.

### XAI modelling of the correction factor, δ

**Fig. 7:**
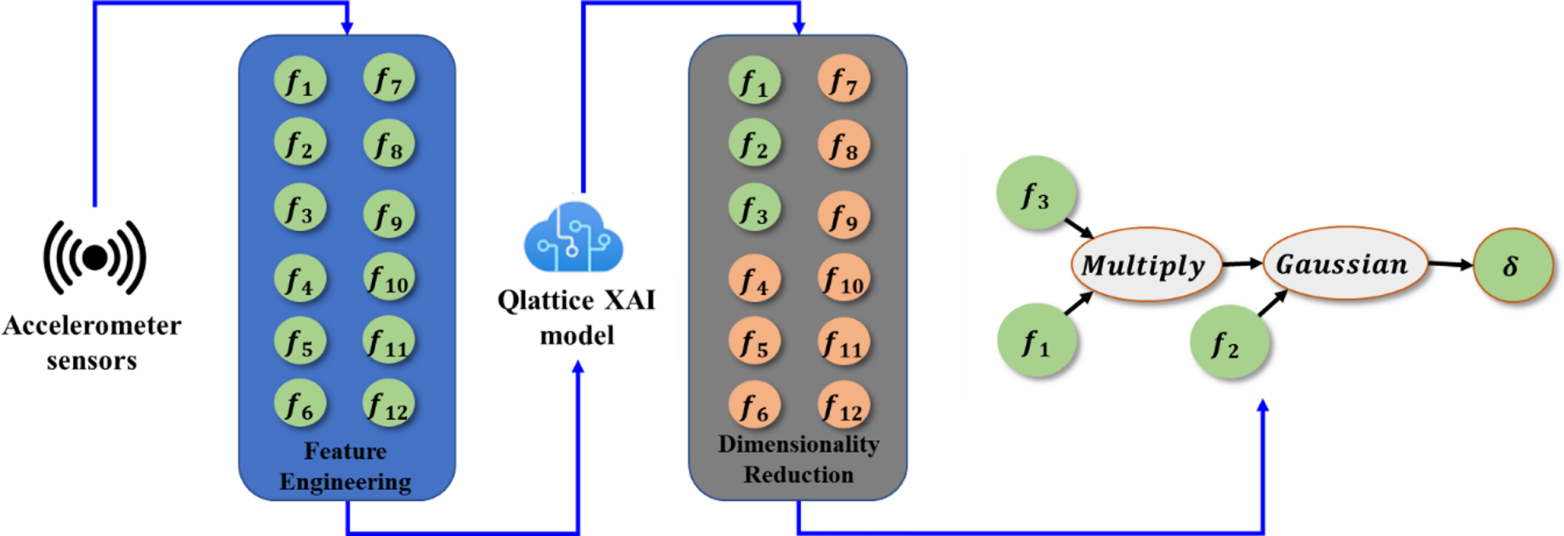
XAI modelling approach

In this step, symbolic regression, one of the most capable XAI approach, is used to model the correction factor based on the extracted raw accelerometer signal features. Feature engineering offers several advantages including simplification, better interpretability and computational efficiency through dimensionality reduction. The input space includes 12 features extracted from the raw accelerometer signals, plus the command frequency, as shown in Table 2. These selected features comprehensively capture all key characteristics of the signal, including its inherent variations and complexities, making it well-suited for robust analysis and model training. The feature set acts as the input to the Qlattice model and the model output is the analytical expression of correction factor *δ.* The overall XAI modelling approach is represented in Fig. 7.

**Table 2:** Extracted features from accelerometer signals

Feature	Label	Mathematical Definitions	Physical interpretations
Command Frequency	f_1_	–	The programmed Z-axis sinusoidal input frequency
Mean Absolute Value (MAV)	f_2_	$\:\text{}\frac{\mathrm{1}}{\mathrm{N}}\text{}{\sum\:}_{\mathrm{i=1}}^{\mathrm{N}}\text{}\left|{\mathrm{a}}_{\mathrm{i}}\right|$	Reflects average vibration magnitude
Variance	f_3_	$\:\frac{1}{N-1}{\sum\:}_{i=1}^{N}{\left({a}_{i}-\stackrel{-}{a}\right)}^{2}$	Represents signal dispersion and energy spread
Peak Amplitude	f_4_	$\:\mathrm{max}\left|{\mathrm{a}}_{\mathrm{i}}\right|$	Tracks maximum instantaneous acceleration
Root Mean Square (RMS)	f_5_	$\:\sqrt{\frac{1}{N}{\sum\:}_{i=1}^{N}{a}_{i}^{2}}$	A measure of vibration intensity
Kurtosis	f_6_	$\:\frac{1}{N}{\sum\:}_{i=1}^{N}{\left(\frac{{a}_{i}-\stackrel{-}{a}}{{\upsigma\:}}\right)}^{4}$	Indicates impulsiveness or peakness of the signal
Skewness	f_7_	$\:\frac{1}{N}{\sum\:}_{i=1}^{N}{\left(\frac{{a}_{i}-\stackrel{-}{a}}{\sigma\:}\right)}^{3}$	Measures symmetry of the signal distribution
Crust Factor	f_8_	$\:\frac{\mathrm{Peak}}{\mathrm{RMS}}$	Higher values indicate high transient peaks
Impulse Factor	f_9_	$\:\frac{\mathrm{Peak}}{MAV}$	Indicates sudden impacts in vibration
Shape Factor	f_10_	$\:\frac{\mathrm{RMS}}{MAV}$	Describes waveform smoothness.
Mean Frequency	f_11_	$\:\frac{\sum\:{f}_{k}P\left({f}_{k}\right)}{\sum\:P\left({f}_{k}\right)}$ Computed from the power spectral density $\:P\left(f\right)$	Represents the central frequency of motion
Total Power	f_12_	$\:{\sum\:}_{k}P\left({f}_{k}\right)$	Measures integrated vibration energy

The twelve features listed in Table 2 were extracted from the raw accelerometer signal a(t) to capture its statistical, spectral, and shape-related characteristics. These features are widely used in vibration analysis, machine condition monitoring, and dynamic signal interpretation. Together, these features capture signal magnitude (MAV, RMS, peak), energy distribution (variance, total power), dynamic behaviour/transient sensitivity (crust factor, impulse factor, kurtosis, skewness), frequency content of machine vibrations (mean frequency), input operating condition (command frequency). These characteristics reflect the nonlinear dynamic response of the tool holder and its deviation from ideal sinusoidal motion.

The Qlattice library allows user-specific selection of operators, loss function, and complexity for symbolic regression in Python. Since the model searches the space of all mathematical expressions, it can be quite computationally demanding. To address this challenge by confining the search space, applicable unary and binary operators are custom defined. All basic mathematical operations (‘+’, ‘-’, ‘/’, ‘$\:\times\:$’) along with standard unary operators (trigonometric, gaussian, log, abs) are selected for this task. The loss function selected is root mean squared error (RMSE). Complexity is defined as the sum of the number of features and the number of operators constituting the final expression, disregarding the constants. The Qlattice model also performs dimensionality reduction during the expression search, selecting only the prominent features and suppressing the others. This is done by penalizing unnecessary operators and inputs through a built-in complexity penalty and selecting only the features that improve the objective function (RMSE). As a result, although twelve features were initially provided, the final symbolic expression used only three features, demonstrating strong feature compression and interpretability.

During the training, the model converged after 11,296 evaluations with a final model complexity of 5 (Fig. 7). The final analytical expression output from the XAI model is given as

12 $$\delta\:=29.4e^k-9.71;\:k=f\left(f_1,f_2,f_6\right)$$

13 $$k=-3980\left(1-0.018f_1\right)^2\left(f_6-0.02\right)^2-\left(0.044f_2-1\right)^2$$

The expression accurately captures the input-output relationship with an R^2^ = 0.945 and RMSE of 1.26. Five-fold cross-validation was performed to assess generalisation and robustness. Across the five folds, the hybrid model achieved a mean R² of 0.9213 ± 0.0178 (95% CI: 0.9057–0.9369), a mean RMSE of 1.4658 ± 0.2588 (95% CI: 1.2389–1.6927), and a mean MAE of 1.1081 ± 0.1712 (95% CI: 0.9581–1.2581). These results indicate that the symbolic regression model demonstrates stable performance with limited sensitivity to data partitioning.

The performance is consistent with the 80/20 hold-out evaluation (R² = 0.945, RMSE = 1.26), confirming strong generalisation capability. Among the 12 features, only 3 features made it to the final expression, demonstrating a significant dimensionality reduction, which is expected to substantially benefit the real-time computational performance.

**Fig. 8:**
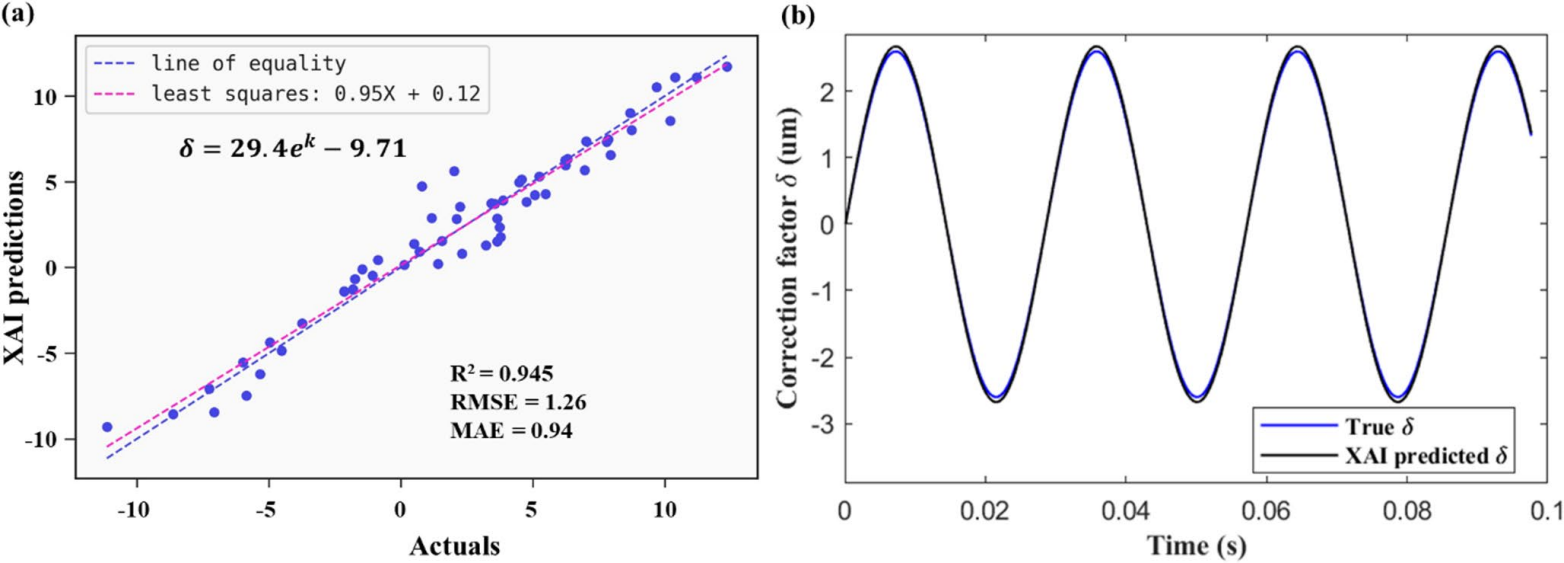
(**a**) Predictive performance of Qlattice (**b**) comparing Qlattice predicted δ with true δ

Symbolic regression reveals the computational structure by presenting an equation, unlike black-box models. Through feature selection, it identifies significant features and their inter-relationships, shedding light on core contributing factors. For instance, based on Eqs. (12) and (13), the frequency, mean absolute value and kurtosis have predominant influence on the correction factor,$\:\:\delta\:$. These insights guide improvements in motion tracking and displacement estimation techniques. The overall predictive performance is shown in Fig. 8 (a). Figure 8 (b) compares the predicted $\:{\delta\:}_{XAI}$ with true δ.

To systematically benchmark the proposed Qlattice-based symbolic regression model, we compared it against several mainstream regression baselines:

- Linear Regression (LR): a standard multiple linear regression model using all input features.
- Lasso Regression: an L1-regularised linear model that promotes sparsity and performs embedded feature selection.
- Decision Tree Regressor (DTR): a single regression tree representing a classical interpretable non-linear model.
- Random Forest Regressor (RF): an ensemble of decision trees trained with bootstrap aggregation, widely adopted as a strong non-linear baseline.

All baseline models were implemented in scikit-learn and trained using the same feature set as Qlattice and the performance comparison is given in Table 3. Qlattice performed better compared to other baseline models. The superior performance of the Qlattice-based symbolic regression model arises from its ability to identify compact, physics-like nonlinear expressions that capture the underlying structure of the correction factor δ. Unlike Lasso regression, which assume fixed linear forms, or decision tree and random forest models, which rely on discontinuous axis-aligned splits, Qlattice searches directly in the space of mathematical expressions and automatically balances accuracy with model complexity. This enables Qlattice to discover smooth, interpretable nonlinear relationships involving only the most relevant features. The selected features (frequency, mean absolute value, and kurtosis) align with the expected physical drivers of dynamic error, further reinforcing the suitability of symbolic regression for modelling δ.

**Table 3:** Performance comparison with other baseline AI models

Model	Key Hyperparameters	*R*²
Lasso Regression	α selected via LassoCV (3-fold), L1 penalty	0.823
Decision Tree Regressor	Max 5 nodes	0.85
	Max 6 nodes	0.91
	Max 8 nodes	0.926
Random Forest Regressor	n_estimators = 200, max_depth = None, random_state = 42	0.814
Qlattice (proposed)	complexity penalty default; 3 selected features	**0.945**

### Performance of hybrid physics-based XAI modelling

The XAI model of correction factor is utilized for latent variable adjustment. In this step, the $\:{\delta\:}_{XAI}$ is combined with decomposed latent variable from physics-based displacement signal ($\:LV{D}_{Ph}$) and finally, the displacement signal is reconstructed.

**Fig. 9:**
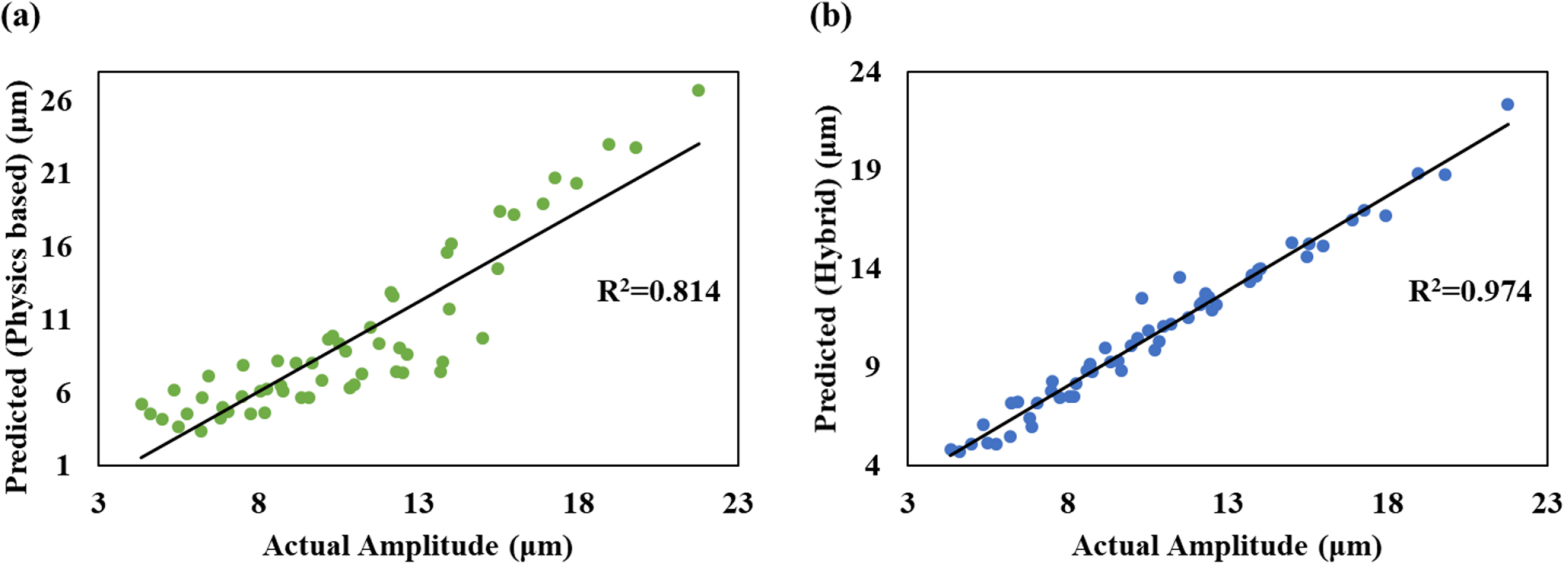
Modelling performance of (**a**) physics-based, (**b**) hybrid physics-based XAI approaches

The predictive performance of the physics based, and hybrid models are compared against the ground truth in Fig. 9. Compared with the physics-based model (RMSE = 2.87), the hybrid physics–XAI model (RMSE = 0.6529) achieves a 77.27% reduction in prediction error computed using the formula:

$$\text{Improvement (\%)}=\frac{{\mathrm{RMSE}}_{\mathrm{phys}}-{\mathrm{RMSE}}_{\mathrm{hybrid}}}{{\mathrm{RMSE}}_{\mathrm{phys}}}\times\:100$$

Also, it is observed that the R^2^ value of hybrid model (R^2^ = 0.974) is 19.65% more than physical model (R^2^ = 0.814). To assess whether the performance gain of the hybrid model was statistically significant, a paired two-tailed t-test was conducted on the sample-wise squared errors of the physics-based and hybrid models in the 80/20 evaluation. The test revealed a highly significant reduction in prediction error with the hybrid approach (*p* = 5.97 × 10⁻⁸). Together with the 77.27% reduction in RMSE, this demonstrates that the hybrid physics–XAI model provides a substantially and statistically superior prediction capability compared with the physics-only baseline. The significant boost is due to the accurate prediction and compensation of physical model error by the XAI.

**Fig. 10:**
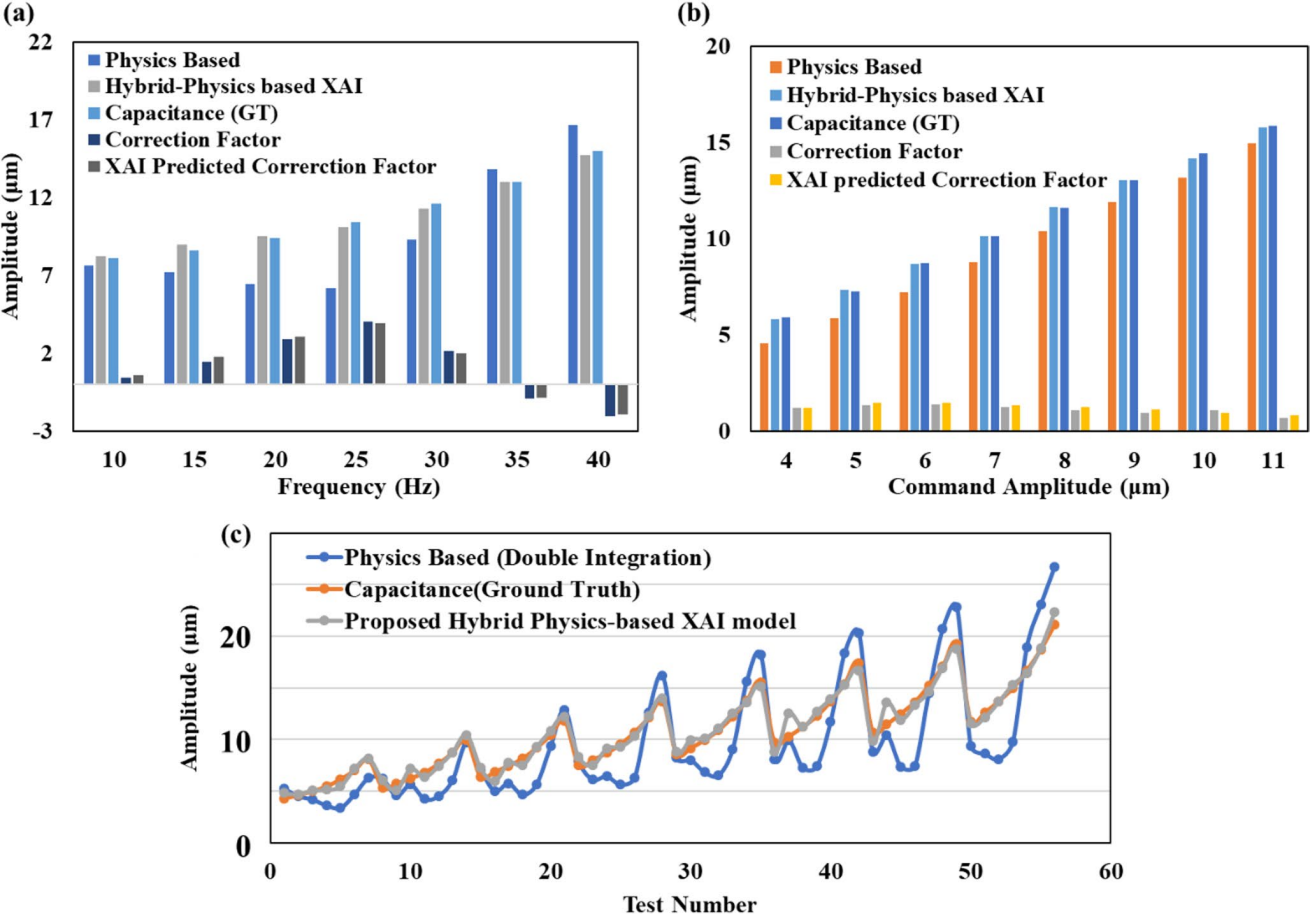
Comparing physical and hybrid model predictions with actual displacement across (**a**) frequencies (**b**) amplitudes (**c**) test numbers

Figure 10 compares the physical and hybrid model displacement amplitudes with ground truth, across frequencies (Fig. 10 (a)) and amplitudes (Fig. 10 (b)). The bar chart demonstrates the capability of the hybrid model to match the ground truth across a range of amplitudes and frequencies. It is worth noting that the peculiar trend of δ with respect to frequencies has been rightly modelled by XAI, enabling accurate predictions using the hybrid approach.

**Fig. 11:**
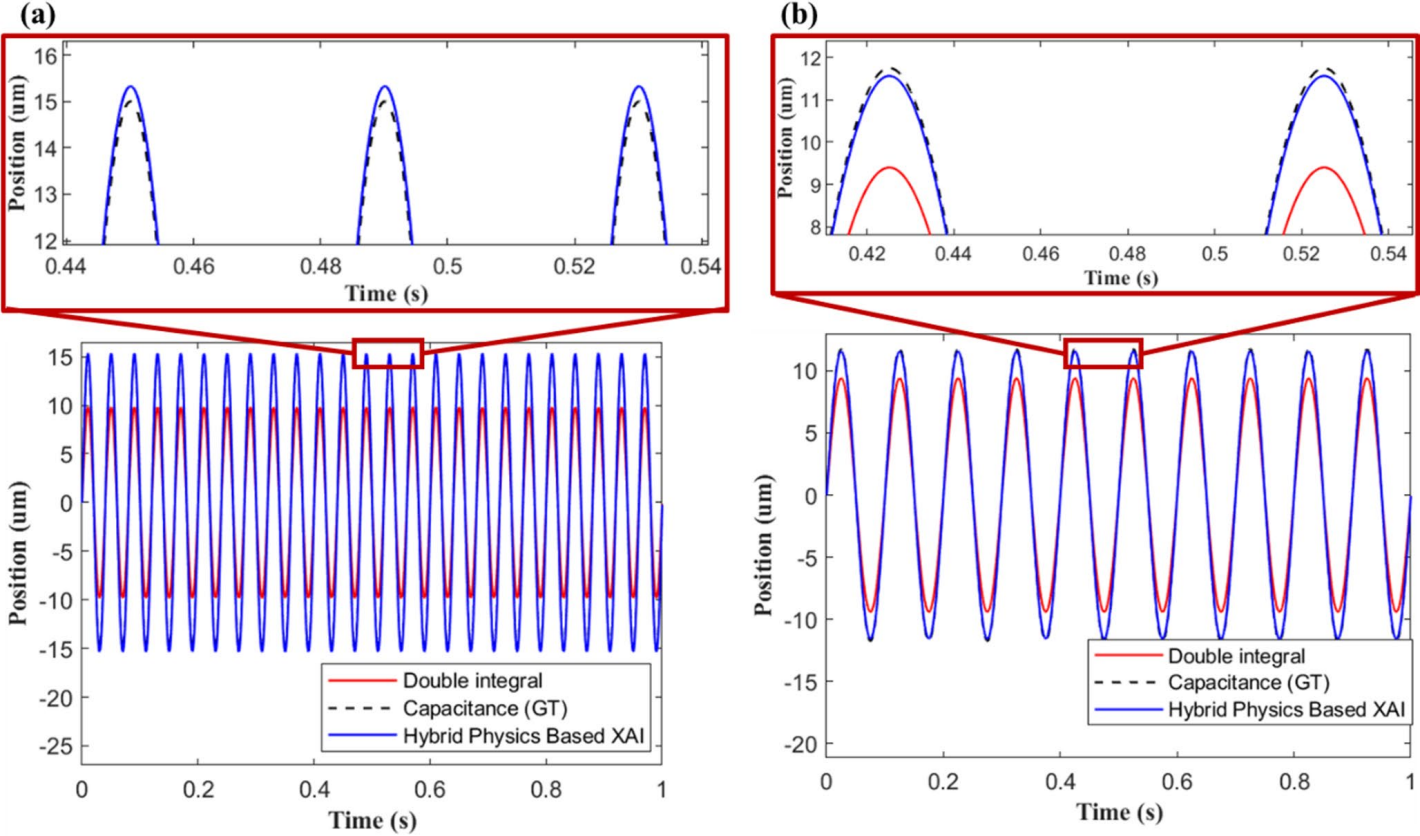
Comparing physical, hybrid and actual position signals at (**a**) 10 Hz and (**b**) 25 Hz

**Fig. 12:**
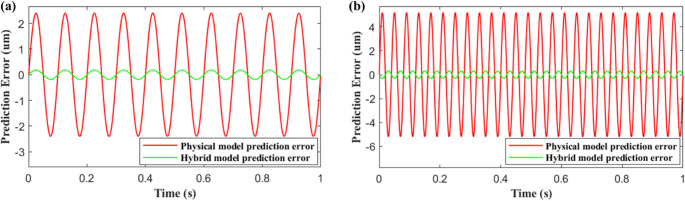
Comparing physical and hybrid modelling errors at (a) 10 Hz and (**b**) 25 Hz

Figure 11 demonstrates the effectiveness of the hybrid approach which can effectively match the true displacement measured by the capacitance probe. The prediction error between the physical and hybrid models is compared in Fig. 12 for frequencies of 10 Hz and 25 Hz respectively. Unlike the case of the physical model, hybrid model errors remained marginal even at higher frequencies, proving its capability to work across a wide range of operating conditions.

## Conclusions

The study proposed a novel and innovative hybrid physics-based XAI modelling approach to mitigate the challenges associated with purely physics based and data-driven modelling. The approach involves modelling the inherent errors of the physics-based model through an XAI approach and subsequently integrating them for an overall enhanced performance. This approach is demonstrated to predict TCP displacement based on accelerometer signals during STS ultra-precision diamond turning operation. The model achieved 19.65% better prediction accuracy during displacement prediction, with an overall R^2^ value of 0.974.

Overall, the proposed hybrid physics-based XAI is shown to be a more advanced and generic modelling approach in smart manufacturing, where the performance of the physics-based model is significantly enhanced without compromising the overall physical interpretability. This approach efficiently combines the interpretability of physics-based models and the flexibility of data-driven approaches, allowing for a more transparent and accurate representation of complex manufacturing processes. Such a synergy could provide valuable insights, promoting better decision-making, potentially enhancing the overall operational efficiency, reducing costs, and improving the part quality.

Looking ahead, this approach could enable the development of next-gen manufacturing digital twins that not only simulate real-world behaviour but also offer actionable insights by integrating domain knowledge and data-driven learning. Future research will also involve testing the model performance on more manufacturing datasets and exploring more XAI techniques.

## Data Availability

All data underpinning this publication will be openly made available at the University of Strathclyde Knowledge Base 10.15129/453da619-3b55-482a-a045-ea93c3edbbae.
